# A Portable System for Remote Rehabilitation Following a Total Knee Replacement: A Pilot Randomized Controlled Clinical Study

**DOI:** 10.3390/s20216118

**Published:** 2020-10-27

**Authors:** Kevin M. Bell, Chukwudi Onyeukwu, Clair N. Smith, Adrianna Oh, Annette Devito Dabbs, Sara R. Piva, Adam J. Popchak, Andrew D. Lynch, James J. Irrgang, Michael P. McClincy

**Affiliations:** 1 Department of Orthopaedic Surgery, School of Medicine, University of Pittsburgh, Pittsburgh, PA 15213, USA; Onyeukwu.Chukwudi@medstudent.pitt.edu (C.O.); CNS45@pitt.edu (C.N.S.); oh.adrianna@medstudent.pitt.edu (A.O.); jirrgang@pitt.edu (J.J.I.); mcclincymp@upmc.edu (M.P.M.); 2 Department of Bioengineering, Swanson School of Engineering, University of Pittsburgh, Pittsburgh, PA 15213, USA; 3 Clinical and Translational Science Institute, University of Pittsburgh, Pittsburgh, PA 15213, USA; 4 Department of Acute and Tertiary Care, School of Nursing, University of Pittsburgh, Pittsburgh, PA 15213, USA; ajdst42@pitt.edu; 5 Department of Physical Therapy, School of Health and Rehabilitation Sciences, University of Pittsburgh, Pittsburgh, PA 15213, USA; spiva@pitt.edu (S.R.P.); ajp64@pitt.edu (A.J.P.); adl45@pitt.edu (A.D.L.)

**Keywords:** interactive health technologies, remote rehabilitation, total knee replacement

## Abstract

Rehabilitation has been shown to improve functional outcomes following total knee replacement (TKR). However, its delivery and associated costs are highly variable. The authors have developed and previously validated the accuracy of a remote (wearable) rehabilitation monitoring platform (*interACTION*). The present study’s objective was to assess the feasibility of utilizing *interACTION* for the remote management of rehabilitation after TKR and to determine a preliminary estimate of the effects of the *interACTION* system on the value of rehabilitation. Specifically, we tested post-operative outpatient rehabilitation supplemented with *interACTION* (n = 13) by comparing it to a standard post-operative outpatient rehabilitation program (n = 12) using a randomized design. Attrition rates were relatively low and not significantly different between groups, indicating that participants found both interventions acceptable. A small (not statistically significant) decrease in the number of physical therapy visits was observed in the *interACTION* Group, therefore no significant difference in total cost could be observed. All patients and physical therapists in the *interACTION* Group indicated that they would use the system again in the future. Therefore, the next steps are to address the concerns identified in this pilot study and to expand the platform to include behavioral change strategies prior to conducting a full-scale randomized controlled trial. Trial registration: ClinicalTrials.gov NCT02646761 “*interACTION*: A Portable Joint Function Monitoring and Training System for Remote Rehabilitation Following TKA” 6 January 2016.

## 1. Introduction

Knee osteoarthritis (OA) is a chronic condition [1] and the leading cause of pain and disability in older adults [2,3]. If conservative treatments do not relieve symptoms, a total knee replacement (TKR) is frequently recommended. In 2010, 719,000 TKR surgeries were performed in the United States and the number of annual TKRs is projected to double or triple over the next 20–30 years [4,5]. TKR represents the highest aggregate cost among rapidly increasing surgical procedures [6,7]. The rapidly increasing number of TKRs and their associated cost threaten to impose an enormous economic burden on the healthcare system in the United States and around the world. In an attempt to combat these rising costs, the Centers for Medicare and Medicaid Services (CMS) introduced the Comprehensive Care for Joint Replacement (CJR) Model in 2016, which proposes a bundled payment model [8]. Unlike fee-for-service payment models, bundled payments incentivize value-based care wherein providers use coordinated, multidisciplinary healthcare approaches to enhance quality, efficiency, and patient satisfaction while controlling costs [9].

Cost analyses have shown that post-acute care accounts for the greatest cost variation in the TKR episode of care [10,11], thus post-acute care is the most logical target for cost savings under the bundled care model [12]. Rehabilitation is a major component of post-acute care with significant cost and relevance to functional outcomes. Rehabilitation has been shown to improve functional outcomes following TKR [13,14,15,16]. However, its delivery and associated costs are highly variable [17]. Inadequate rehabilitation can lead to longer recovery, further injury from improper performance, and increased downstream costs, so its costs must be minimized while ensuring its effective delivery [18,19].

The current status quo of physical therapy delivery has notable limitations. A nationwide shortage of physical therapists is projected, limiting in-person access as demand increases [20]. Access is further impaired by mobility and income limitations such as transportation issues, inability to handle copays, and low health literacy [21,22,23,24,25]. Even in optimal settings, the majority of rehabilitative work is done through home exercise. However, non-adherence with the prescribed home exercise regimen may be as high as 70% [26,27,28] and the factors contributing to non-adherence are multifactorial. Early evidence suggests that tele-rehabilitation and/or remote rehabilitation using mobile health technologies has the potential to overcome these barriers and improve access, accountability, and value [29,30,31,32,33,34,35,36,37,38,39].

The authors have developed and previously validated the accuracy [40] of a remote rehabilitation monitoring platform (*interACTION*) that uses portable inertial measurement units (IMUs) combined with a mobile application and back-end clinician portal. The present study’s objective was to assess the feasibility of utilizing *interACTION* for remote management of rehabilitation after TKR and to determine a preliminary estimate of the effects of the *interACTION* system on the value of rehabilitation. Value was operationally defined as the change in the Activities of Daily Living Scale (ADLS) of the Knee Outcome Survey divided by the cost of rehabilitation. Specifically, we tested post-operative outpatient rehabilitation supplemented with *interACTION* by comparing it to a standard post-operative outpatient rehabilitation program using a randomized design.

## 2. Materials and Methods

### 2.1. Overview

This study has been registered with ClinicalTrials.gov (NCT02646761). This was a two-group pilot randomized clinical study implemented from June 2016 to September 2017 in the UPMC Centers for Rehab Services (CRS, Pittsburgh, PA, USA). We enrolled male and female subjects between the ages of 40 and 80 years who were scheduled to undergo a primary unilateral TKR and who had been referred for post-operative outpatient physical therapy at a UPMC CRS facility. Exclusion criteria were bilateral or revision TKR; patients who had a pre-operative referral to a skilled nursing facility or were transferred to a skilled nursing facility for post-operative care; patients with BMI >40 at the time of surgery; individuals who were not free of any other co-disability or comorbidity that would specifically hinder performance of rehabilitation exercises; and individuals who could not understand/utilize the audio and visual feedback from the system.

### 2.2. Recruitment and Consent

Subjects scheduled to receive a TKR at a UPMC facility were recruited pre-operatively at Orthopedic Surgery clinics or the pre-operative educational session that is required for all patients. The research coordinator fully explained the study, checked for eligibility, and then recruited patients. The University of Pittsburgh Institutional Review Board approved the study protocol (PRO15060281). All participants provided written informed consent before conducting any research procedures.

### 2.3. Randomization

The randomization sequence was computer-generated in block sizes of 4, 6, and 8. Allocation was sealed in opaque and consecutively numbered envelopes. The research coordinator pre-operatively randomized to one of two groups: standard post-operative outpatient rehabilitation (Control Group) or *interACTION* Group.

### 2.4. Interventions

In the Control Group, patients performed their post-operative outpatient rehabilitation in the clinic with a physical therapist. Patients visited/attended the clinic for two to three sessions per week, over a maximum of 10 weeks and supplemented with a home exercise program. To help maximize generalizability, physical therapists were asked to treat the patient according to the standard “best practices” for post-operative outpatient rehabilitation.

In the *interACTION* Group, post-operative outpatient rehabilitation was performed in the clinic with a physical therapist and supplemented with *interACTION* to remotely manage the home exercise program. For the first two weeks, patients used *interACTION* during the routine outpatient rehabilitation clinic sessions to familiarize themselves with the system. Patients were then issued an *interACTION* device for home use. Over eight weeks, participants in the *interACTION* Group continued to return to the outpatient rehabilitation facility but were asked to reduce their number of weekly visits to the outpatient facility by one visit per week while concurrently using *interACTION* at home.

### 2.5. Device Description

**Figure 1:**
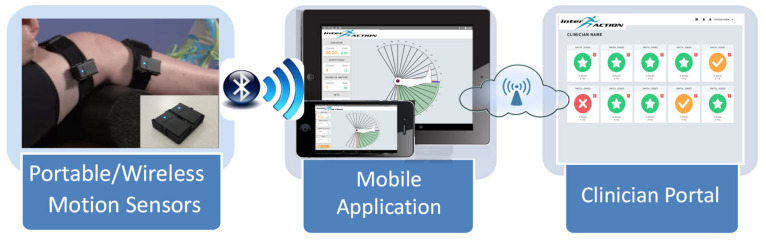
Conceptual flowchart of the *interACTION* platform: (1) inertial measurement units (IMUs) attached to the patient’s thigh and shank using neoprene straps and 3D-printed cases, (2) mobile application utilized by the patient to complete their prescribed home exercises, (3) screenshot of clinician portal depicting the patient dashboard which is utilized by the physical therapist to manage the patient’s rehabilitation remotely.

*InterACTION* (University of Pittsburgh Ref: 03425, 04275) utilizes portable motion sensors placed on either side of a joint and attached with adjustable neoprene straps (Figure 1–Left), to collect joint orientation data using a custom mobile application (Figure 1–Middle). A more thorough description of the technical parameters of the motion sensors has been published previously [36]. The mobile application contains 30 knee-specific home exercises for TKR rehabilitation. The physical therapist creates a personalized home exercise program from these exercises and can offer remote guidance through the clinician portal (Figure 1–Right).

A written description and video demonstration are provided for each exercise. Before the patient performs their exercises, the mobile application guides them through a series of recovery metrics to assess their progress. The patient then uses the interface to complete the prescribed exercises. The motion data are analyzed to provide real-time feedback to the patient.

The feedback utilizes on-screen animation of knee motion and a color-coded feedback mechanism to inform the patients if they are outside (red), approaching (yellow), or have reached (green) the target range of motion for their exercise. When the feedback indicator turns yellow, the system automatically increments the “attempted repetition counter” and when the indicator turns green the system automatically increments the “successful repetition counter.” This two-tiered approach allows the physical therapist to determine if the patient performed the exercises correctly.

The adherence and recovery data collected in the mobile application are automatically synchronized, using a wireless connection, to a web-based clinician portal. The clinician portal utilizes a dashboard to enable the physical therapist to monitor multiple patients simultaneously and identify patients needing personalized attention. The physical therapist can remotely refine a treatment plan using the portal, which automatically synchronizes with the mobile application used by the patient. The clinician portal also has built-in communication options that enable the physical therapist to contact the patient directly.

### 2.6. Clinical Measures

Outcome assessment was carried out by the research coordinator who was not blinded to the group assignment. Assessments were performed at baseline (first post-operative outpatient rehabilitation visit) and at 5 weeks (±7 days) and 10 weeks (±7 days) from baseline.

The feasibility of the interventions was assessed by adherence to the home exercise sessions (Control Group), device usage (*interACTION* Group), loss to follow-up, and attrition. Control Group participants were asked to record their daily performance of the prescribed home exercises on a paper-based exercise log. *InterACTION* Group participants were asked to use *interACTION* daily to guide them through their home exercise program.

The primary outcome was value, which was operationally defined as the change in the Activities of Daily Living Scale (ADLS) of the Knee Outcome Survey at 10 weeks divided by the total cost of rehabilitation. The ADLS is a 14-item measure of symptoms and activity limitations for individuals with a variety of knee impairments [41,42]. The cost of rehabilitation was determined based on the billing records of the CRS physical therapy facility, which is a function of the total number of physical therapy sessions and the billable charges for each session during the 10 weeks the patients were enrolled in the study.

The following secondary outcome measures were collected at baseline and 5-week and 10-week follow-ups: active range of motion (aROM) [43], in which ROM was calculated as the difference between maximum flexion and maximum extension, measured with a standard goniometer while the patient lies in the supine position; the Numeric Pain Rating Scale (NPRS), which asks participants to rate their pain levels currently and their level of worst and least pain in the past 24 h on a scale from 0 (no pain) to 10 (worst pain imaginable); and the Veterans RAND 12-Item Health Survey (VR-12), a 12-item patient-reported general measure of health-related quality of life [44].

Functional performance was tested in all patients at the 5-week and 10-week timepoints using a battery of four performance-based tests: (1) the six-minute walk test [45,46], which assesses the distance the subject is able to walk over a period of six minutes; (2) the stair climbing test [47], in which a patient ascends and descends a standard flight of stairs as quickly and safely as possible; (3) the timed up and go test (TUG) [48], in which the patient sits in a standard armed chair, rises, walks three meters, turns around, walks back to the chair, and sits; and (4) the unilateral balance test [45,46], in which the patient attempts to maintain single leg balance for up to two minutes. The functional performance tests were not performed at baseline due to the fact that the baseline testing occurred too close to surgery.

At the 10-week assessment, the research coordinator conducted a semi-structured interview with all patients in the *interACTION* Group and their physical therapists to query about their experiences with rehabilitation and the *interACTION* platform via a combination of survey questions, a self-reported assessment of adherence, and open-ended discussion questions.

### 2.7. Sample Size Determination

Given the nature of the funding source and timeframe for completion of the study, the sample size was limited to a total of 60 subjects (30 per group). Assuming a one-tailed test with an alpha of 0.05, a total sample size of 60 patients (30 per group) enabled us to detect a medium to large effect (standardized mean difference of 0.65) in value. The statistical justification presented is based on historical data, which may or may not be reflective of the population recruited in this study.

### 2.8. Statistical Analyses

A modified intention-to-treat analysis was implemented in this study. No data were excluded from the analysis based on a participant’s level of adherence or device usage. However, only participants who completed baseline testing were included in the analysis. All data that were available for all participants were included in the analysis, and no attempt was made to approximate the missing data points.

A Wilcoxon rank-sum test was used to compare the primary outcome (value) measures between groups. The alpha value was set at α = 0.05 for all outcomes. A Wilcoxon rank-sum test was also used to compare cost analyses parameters (weeks of physical therapy, number of physical therapy visits, total time, and total cost) between the two groups.

For the feasibility analysis, adherence and device usage were calculated as the percentage of the total number of exercise sessions recorded between weeks 3 through 10 relative to a total of 40 possible exercise sessions over the 8-week period (assuming 5 sessions per week). Loss to follow-up was calculated as the number of participants randomized to each group minus the number of participants that completed the baseline assessment. Attrition was calculated as the number of participants that completed baseline testing, but did not complete the 10-week assessment, divided by the number of subjects that completed the baseline assessment.

For the ADLS and aROM (flexion), a repeated measures ANOVA or a two-sample *t*-test was used to compare differences between groups if the distribution was normal. A Wilcoxon rank-sum test was used for non-normally distributed data.

The functional performance tests were assessed at the 5- and 10-week visits, using an averaged z-score (or standard score) to calculate a cumulative score for the six-minute walk (distance in feet), stair climbing (time divided by steps), TUG (time), and unilateral balance (time) tests [49]. On average, the z-scores were close to zero, meaning the scores for each individual subject did not differ from the mean score across all subjects. Differences between the cumulative z-scores were assessed using a repeated measures ANOVA or a two-sample *t*-test was used to compare differences between groups if the distribution was normal. A Wilcoxon rank-sum test was used for non-normally distributed data.

The semi-structured patient and physical therapist interviews were transcribed and analyzed by a qualitative data analysis team (Qualitative, Evaluation and Stakeholder Engagement [Qual EASE] Research Services, Center for Research on Health Care Data Center, University of Pittsburgh) unaffiliated with the research team, to minimize the potential for bias. The Qual EASE team developed a qualitative coding scheme that enabled all responses to be categorized as criticisms, affirmations, or suggestions. The qualitative coding was then completely co-coded and fully adjudicated by two independent Qual EASE analysts.

## 3. Results

**Figure 2:**
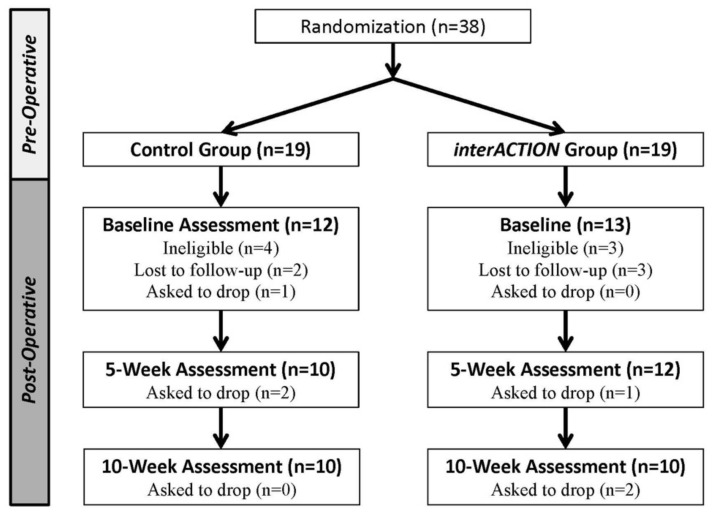
Consolidated standards of reporting trials (CONSORT) diagram of participant flow during the study.

We screened 47 people for participation in this study. Nine were deemed ineligible and 38 individuals were randomized (mean [SD] age; 64.4 [8.2], 48% female) (Figure 2). Of the 19 individuals randomized to the Control Group and 19 randomized to the *interACTION* Group, 12 individuals in the Control Group and 13 in the *interACTION* Group completed baseline assessment post-operatively. Seven participants in the Control Group and six in the *interACTION* Group were unable to complete baseline testing after randomization. Attrition did not differ between the groups at 10 weeks: values were 16.6% (2 of 12) in the Control Group and 23.0% (3 of 13) in the *interACTION* Group. Baseline characteristics were similar between groups (Table 1).

### 3.1. Value

The primary outcome was value—calculated as the ratio of change in ADLS from baseline to 10-week follow-up divided by the total cost of physical therapy. Considering the parameters individually, no difference could be detected in delta ADLS (Control: 33.6 [13.9], *interACTION*: 30.5 [13.1], *p* = 0.555) or total cost (Control: USD 2231 [USD 1255], *interACTION*: USD 2452 [USD 1160], *p* = 0.67) (Table 2). Similarly, for value (delta ADLS/total cost), no difference could be detected between groups (Control: 0.018 [0.009], *interACTION*: 0.013 [0.007], *p* = 0.32].

### 3.2. Adherence

One of the primary goals of *interACTION* was to increase adherence to home exercise. During the 10-week semi-structured interviews, all participants were asked to self-report their level of adherence. One participant in the control group failed to complete the self-reported adherence question, reducing the sample size n = 9. Self-reported rates of adherence were similar between groups, with five out of nine individuals in the Control Group reporting >90% adherence and 6 out of 10 individuals in the *interACTION* Group reporting >90% adherence (Table 3). Participants in the Control Group also completed a paper-based exercise log, which showed 50% [37%] adherence over the 10-week period. Participants in the *interACTION* Group were not asked to complete an exercise log. Device usage was calculated as a surrogate measure of adherence and determined to be 37% [24%] between week 3 and week 10.

### 3.3. Secondary Outcomes

The study was not powered to detect differences in the secondary clinical and functional performance outcome measures. However, the feasibility of collecting the secondary outcomes at baseline, 5 weeks, and 10 weeks was established in this pilot study with no more than one missing data point observed for any of the secondary clinical or functional performance outcome measures at any time point (Table 4). Preliminary analysis showed no detectable significant differences for any of the secondary outcome measures at any time point, with the exception of NPRS within the last 24 h, when measured at 10 weeks, which indicated higher pain levels in the *interACTION* Group compared to the Control Group (Control: 2.2 [2.5], *interACTION*: 3.7 [1.9], *p* = 0.043).

### 3.4. Survey Results

Analysis of the patient survey questions showed that three of the seven survey questions had a median score of 4 and the remaining four survey questions had a median score of 3 (Table 5). The sample size for the patient surveys was n = 11, rather than n = 10 as indicated in Figure 2, due to the fact that one of the participants that asked to be dropped from the study prior to the 10-week assessment still completed a survey. Generally, the majority of patients found the exercises relevant to their rehabilitation (median = 4, interquartile range [IQR] = 1), the visual feedback easy to understand (median = 4, IQR = 2), and forms of media (videos, text, motion feedback, etc.) useful (median = 4, IQR = 1). All eleven patients surveyed said that they would consider using *interACTION* in the future.

Of the n = 10 physical therapists in the *interACTION* group whose patients completed the 10-week assessment, n = 9 were interviewed and n = 6 completed the survey. Analysis of the physical therapist surveys showed that six of the ten survey questions had a median score of 4 and the remaining four survey questions had a median score of 3 or 3.5. Additionally, all six physical therapists surveyed indicated that they would consider using *interACTION* to manage rehabilitation again in the future (Table 6).

Qualitative analysis of the open-ended discussion questions indicated that answers from the patients and physical therapists frequently overlapped in terms of what they liked (affirmations), did not like (criticisms), and what they would like to see changed or improved (suggestions), with the *interACTION* system. The criticisms focused on the functionality of the equipment and the interface, while the affirmations focused on motivation and accountability, as well as the system’s potential for future use.

The results of the patient interviews are summarized in Table 7. The sample size for the patient interview qualitative analysis was limited to n = 7 due to errors in the audio recording procedures. In total, 34 quotations across the seven patient interviews were coded as affirmations. With a few exceptions, patients felt that using the *interACTION* system or participating in the study gave them additional motivation or accountability to do their exercises. Specifically, six patients indicated that *interACTION* gave them motivation/accountability and four patients indicated that they found the visual feedback useful.

Thirty-eight quotations across the seven patient survey interviews were coded as criticisms. The main complaints were calibration and sensor issues and mobile application failures. Patient suggestions were more diverse than the criticisms or affirmations, though lower in occurrence, at 13 quotations counted across the seven transcripts. Suggestions included improving the user interface and the sensor harnesses and straps.

The number of affirmations (31 quotations) was roughly the same as the criticisms (29 quotations) across the nine provider interviews (Table 8). There was a wide variety of affirmations provided by the physical therapists, with five providers indicating that *interACTION* provided motivation/accountability and five indicating that the reports and tracking are useful. The most common criticism from providers was that the calibration or counting errors led to inconsistent measurements of range of motion, which frustrated the patients. There were fewer quotations in the provider interviews coded as suggestions than there were criticisms and affirmations, with only 10 coded across the nine transcripts. However, the number of unique suggestions was roughly commensurate with the unique complaints and points of praise. The suggestions made by the physical therapists could generally be described as addressing their own criticisms. The two most common suggestions were to improve the calibration of the sensors and simplify the setup for patients.

## 4. Discussion

This study assessed the feasibility of utilizing *interACTION* for remote management of rehabilitation after TKR and estimated the effects of the *interACTION* system on the “value” of rehabilitation. Specifically, we tested post-operative outpatient rehabilitation supplemented with *interACTION* by comparing it to a standard post-operative outpatient rehabilitation program using a randomized design.

The original sample size estimation indicated that we needed to recruit a total of 60 subjects (30 per group). However, due to the nature of the funding source and the timeframe for completion of the study, recruitment was terminated after 38 subjects were enrolled. Feedback from participants in the *interACTION* group, both informally throughout the study period and during the 10-week semi-structured interviews, indicated that although participants were very optimistic about the concept and potential for *interACTION*, they experienced functionality and usability issues that hindered the impact of the intervention. Therefore, the decision was made to stop recruitment before originally planned, and utilize the feedback from the *interACTION* participants as a component (field test) of a user-centered design process [50]. A sample size of 38 was sufficient to assess the feasibility of the study design. Preliminary analysis of the primary and secondary clinical outcomes was also conducted and presented.

Baseline feasibility characteristics were similar between groups, indicating that the computer-generated randomization scheme was effective. The attrition rates were relatively low and not significantly different between groups, indicating that participants found both interventions acceptable. We did observe a relatively high loss to follow-up between recruitment and baseline testing. The primary reason for this loss was that participants were no longer eligible (7 of 13), because they either went to a skilled nursing facility post-operatively or elected to receive physical therapy at a non-UPMC CRS facility. An additional 5 of the 13 patients could not be contacted post-operatively. This study design limitation should be addressed in future trials by revising the exclusion criteria so that recruitment begins post-operatively to eliminate the gap in contact, or by beginning baseline testing and intervention pre-operatively to ensure that patients engage with the platform prior to surgery.

Additionally, the participants in the *interACTION* Group were asked to reduce the number of weekly visits by one visit per week after their two-week training period. This instruction was ultimately left to the discretion of the physical therapist and patient. Although a small (not statistically significant) decrease in number of physical therapy visits was observed in the *interACTION* Group, the effect was not as large as anticipated and no significant difference in total cost could be observed. It was unexpected that the average costs in the *interACTION* Group were slightly higher than the Control Group. However, subsequent analysis of the individual CPT codes revealed a single outlier in each group that accounted for this difference. As a result, no difference in the value, the primary outcome, could be detected. After feasibility and efficacy have been established, future cost-effectiveness trials should place tighter restrictions on the number of visits if value is the primary outcome.

We were unable to detect any consistently significant differences in the secondary clinical and functional performance outcomes measures, with the exception of NPRS within the last 24 h, which was higher at 10 weeks in the *interACTION* Group. Future work is required to determine if this observation is a result of the small sample size or if the *interACTION* intervention negatively impacted pain levels.

To date, the most directly comparable studies were published by Piqueras et al. [29] and Correia et al. [30]. Piqueras et al. evaluated the effectiveness of a virtual telerehabilitation system, comprising nine degree-of-freedom wireless kinematic sensors, in patients after total knee replacement [29]; the primary outcome was function, assessed with the active range of knee movement. They determined that a two-week interactive telerehabilitation program was at least as effective as conventional therapy. Similarly, Correia et al. presented a home-based rehabilitation platform that included digital biofeedback using inertial motion trackers to digitize patient motion and provide real-time feedback on performance through a mobile application [30]. They completed an eight-week feasibility study, using TUG as the primary outcome, and concluded that their digital rehabilitation solution achieved better outcomes than conventional in-person rehabilitation. Several studies have explored the effectiveness of traditional telerehabilitation [31,32] using activity monitors [33,34,35] or other motion sensing technologies to facilitate the delivery of home exercises [36,37,38,39,51].

We conducted semi-structured interviews with the physical therapists and patients assigned to the *interACTION* Group at the 10-week assessment. Qualitative analysis of the participants enabled us to evaluate their experiences with the *interACTION* platform and to identify areas for improvement. Criticisms seemed to coalesce around a few central themes. Both patients and providers noted that the functionality and usability of *interACTION* could be improved. Patients became frustrated and even demotivated when they experienced connectivity issues or when the sensors’ readings were variable, especially in regard to exercise repetition counts. Some providers felt the system did not give them reliable compliance information.

Suggestions from both groups corresponded with their criticisms and included fixing technical problems, improving hardware functionality, and increasing features and options. The more novel suggestions focused on the desire to expand the technology to empower the patient to take a more active role in their own recovery. Therefore, future work should explore the possibility of incorporating behavioral change strategies that have been shown to be effective in promoting adherence and sustaining behavioral change into the platform [52,53,54,55,56].

Patients and providers also generally agreed, with few exceptions, that if the complaints were addressed and suggestions carried out in a future iteration, *interACTION* would be very useful on both ends of care. Therefore, the next steps are to address the functionality and usability concerns identified in this pilot study and to expand the platform to include behavioral change strategies prior to conducting a full-scale randomized trial to evaluate the utility of the platform.

## Figures and Tables

**Table 1:** Participant characteristics at baseline.

	Total	Control	*interACTION*
	(n = 38)	(n = 19)	(n = 19)
**Age (mean** **± SD)**	64.4 ± 8.2	65.3 ± 8.3	64.0 ± 7.7
**BMI (mean** **± SD)**	31.0 ± 5.2	31.1 ± 5.2	31.8 ± 5.7
**Female**	18 (47%)	9 (47%)	9 (47%)
**Race**			
** White**	32 (84%)	16 (89%)	16 (89%)
** Black**	5 (13%)	2 (11%)	3(16%)
** ** **Hispanic ethnicity**	1 (3%)	1 (5%)	0 (0%)
**Married**	25 (66%)	11(58%)	14(74%)
**Education**			
** Some high school**	1 (3%)	1 (5%)	0 (0%)
** High school grad**	8 (21%)	4 (21%)	4 (21%)
** Some college**	7 (18%)	6 (32%)	1 (5%)
** College graduate school**	10 (26%)	4 (21%)	6 (32%)
** Some graduate school**	3 (8%)	2 (11%)	1 (5%)
** Graduate degree**	9 (24%)	2 (11%)	7 (37%)
**Employment status**			
** Retired due to health**	4 (10%)	2 (11%)	2 (11%)
** Retired not due to health**	17 (44%)	8 (42%)	9 (47%)
** Temporary retired due to health**	3 (8%)	1 (5%)	2 (11%)
** Semi-retired**	1 (3%)	0 (0%)	1 (5%)
** Regular full time**	11 (29%)	7 (37%)	4 (21%)
** Regular part time**	2 (5%)	1 (5%)	1 (5%)
**Current Smoker**	4 (10%)	3 (16%)	1 (5%)

**Table 2:** Value analysis.

	Control	*interACTION*	*p*-Value
	(n = 10)	(n = 10)	
**Weeks of PT**	8.1 ± 3.2	7.3 ± 2.3	0.48
**Number of PT visits**	13.3 ± 5.5	12.7 ± 5.1	0.71
**Total time (minutes)**	786 ± 411	828 ± 394	0.69
**Total cost**	$2231 ± $1255	$2452 ± $1160	0.67
**Delta ADLS/# visits**	3.0 ± 1.6 (8)	2.4 ± 1.0 (8)	0.45
**Value (Delta ADLS/total cost)**	0.018 ± 0.009 (8)	0.013 ± 0.007 (8)	0.32

**Table 3:** Self-reported compliance.

How Compliant Were You on Average with the Rehabilitation Process?			
**(%)**	Total	InterACTION	Control
	(n = 19)	(n = 10)	(n = 9)
**>90%**	11 (61%)	6 (60%)	5 (63%)
**71–90%**	7 (33%)	4 (40%)	3 (25%)
**51–70%**	0 (0%)	0 (0%)	0 (0%)
**31–50%**	1 (6%)	0 (0.0)	1 (13%)
**0–30%**	0 (0%)	0 (0%)	0 (0%)

**Table 4:** Clinical and physical function outcomes over time in study arms (*t*—two-sample *t*-test, *w*—Wilcoxon rank-sum test, *—*p* < 0.05). The sample size for each outcome is also provided (n), with any reduced sample size compared to Figure 2 indicating missing data. The functional performance outcomes were not measured at baseline because the timepoint was too close to surgery.

	Control	(n)	*interACTION*	(n)	*p*-Value
**ADLS**					
** Baseline**	48.7 ± 17.5	(12)	46.5 ± 11.6	(11)	0.835
** ** **5-Week**	70.7 ± 15.3	(10)	66.7 ± 14.8	(12)	0.559
** ** **10-Week**	83.1 ± 13.8	(9)	76.9 ± 13.9	(10)	0.474
** Delta (10-Week – Baseline)**	33.6 ± 13.9	(9)	30.5 ± 13.1	(10)	0.555
**VR-12**					
** Baseline**	27.1 ± 7.8	(11)	26.3 ± 12.1	(12)	0.923
** ** **5-Week**	35.9 ± 7.8	(10)	39.4 ± 10.3	(11)	0.457
** ** **10-Week**	43.5 ± 9.0	(10)	42.2 ± 5.8	(9)	0.898
** Delta (10-Week – Baseline)**	17.4 ± 11.7	(10)	16.0 ± 12.6	(9)	0.972
**Numeric Pain Rating Scale (NPRS) 24 Hours**					
** Baseline**	5.7 ± 3.1	(12)	5.6 ± 2.4	(13)	0.963 *^t^*
** ** **5-Week**	3.0 ± 2.2	(10)	3.1 ± 1.7	(12)	0.920 *^t^*
** ** **10-Week**	2.2 ± 2.5	(10)	3.7 ± 1.9	(10)	0.043 *^w^*
** Delta (10-Week – Baseline)**	−3.5 ± 3.0	(10)	−2.2 ± 2.5	(10)	0.308 *^t^*
**Active Extension (degrees)**					
** Baseline**	1.1 ± 3.4	(12)	5.3 ± 5.6	(13)	0.067 *^w^*
** ** **5-Week**	1.7 ± 3.0	(9)	2.1 ± 2.7	(12)	0.773 *^w^*
** ** **10-Week**	1.7 ± 2.5	(10)	1.6 ± 2.5	(10)	0.878 *^w^*
** Delta (10-Week – Baseline)**	0.6 ± 3.5	(10)	−3.8 ± 5.5	(10)	0.063 *^w^*
**Active Flexion (degrees)**					
** Baseline**	91.5 ± 19.3	(12)	93.0 ± 17.6	(12)	0.810
** ** **5-Week**	119.7 ± 5.1	(9)	116.7 ± 12.0	(12)	0.577
** ** **10-Week**	123.1 ± 4.9	(10)	120.8 ± 8.6	(10)	0.737
** Delta (10-Week – Baseline)**	31.4 ± 21.8	(10)	31.4 ± 20.0	(10)	0.645
**Unilateral Balance Test (seconds)**					
** ** **5-Week**	34.4 ± 36.3	(10)	30.0 ± 28.8	(12)	0.770 *^w^*
** ** **10-Week**	50.0 ± 35.4	(10)	39.7 ± 42.9	(10)	0.256 *^w^*
** Delta (10-Week – 5-Week)**	15.6 ± 28.6	(10)	13.7 ± 34.6	(10)	0.577 *^w^*
**Timed Up and Go (seconds)**					
** ** **5-Week**	9.7 ± 3.1	(10)	8.9 ± 3.2	(12)	0.562
** ** **10-Week**	8.6 ± 2.5	(10)	8.6 ± 3.1	(10)	0.939
** Delta (10-Week – 5-Week)**	−1.1 ± 1.7	(10)	−0.4 ± 2.4	(10)	0.482
**Stair Climb Test (steps/second)**					
** ** **5-Week**	0.6 ± 0.2	(9)	0.6 ± 0.3	(11)	0.606
** ** **10-Week**	0.7 ± 0.3	(9)	0.8 ± 0.4	(10)	0.855
** Delta (10-Week – 5-Week)**	0.2 ± 0.2	(9)	0.1 ± 0.2	(10)	0.646
**6 Minute Walk (meters)**					
** ** **5-Week**	384.8 ± 93.0	(10)	410.1 ± 145.1	(12)	0.820 *^w^*
** ** **10-Week**	445.8 ± 86.6	(10)	478.4 ± 182.6	(9)	0.904 *^w^*
** Delta (10-Week – 5-Week)**	60.9 ± 56.5	(10)	46.4 ± 56.3	(9)	0.582 *^t^*
**Composite Functional Performance (z-score)**					
** ** **5-Week**	0.004 ± 0.701	(10)	0.006 ± 0.932	(12)	0.995 *^t^*
** ** **10-Week**	0.028 ± 0.636	(10)	−0.071 ± 1.021	(10)	0.797 *^t^*
** Delta (10-Week – 5-Week)**	−0.002 ± 0.836	(10)	0.099 ± 0.851	(10)	

**Table 5:** Patient survey responses (n = 11).

Patient Survey Responses (n = 11)	0	1	2	3	4	Median	IQR
Were the exercises in *interACTION* relevant to your rehabilitation? (4 – very relevant)	0	1	0	3	7	4	1
Does the screen layout and the structure of the app make it easy to navigate? (4 – easiest)	0	0	2	6	3	3	0.5
Is it easy to setup and calibrate the sensors for use? (4 – very easy)	1	1	1	3	5	3	1.5
Is the visual feedback from the app easy to understand? (4 – very easy to understand)	0	1	3	1	6	4	2
Are the forms of media (videos, text, motion feedback, etc.) useful? (4 – very useful)	1	0	1	2	7	4	1
Are the angles shown during the exercises helpful in keeping track of your recovery? (4 – very helpful)	2	0	2	2	5	3	2
Is indicating your pain levels helpful in keeping track of your recovery? (4 – very helpful)	3	0	2	3	3	3	2.5
						**Yes**	**No**
Would you consider using the *interACTION* device if you had to do rehabilitation again in the future?						11	0

**Table 6:** Provider survey responses (n = 6).

Provider Survey Responses (n = 6)	0	1	2	3	4	Median	IQR
Are all the exercises provided in the *interACTION* system relevant and useful for rehabilitation? (4 - most relevant)	0	0	1	2	3	3.5	1
Does the screen layout and the calendar view make the clinician portal easy to navigate? (4 - easiest)	0	0	0	1	5	4	0
Is it easy and convenient to modify and assign exercises to the patient? (4 - very easy)	0	0	1	2	2	3	1
Is the visual feedback on the patient’s progress easy to understand? (4 - very easy to understand)	0	0	0	1	4	4	0
Are the forms of media (Patient Summary, Calendar View, Graphs, etc.) used to review data on patient performance and compliance useful? (4 - very useful)	0	0	0	1	4	4	0
Is the “Recovery Score” useful or helpful to keep track of patient recovery? (4 - very helpful)	0	0	0	3	2	3	1
Is the “Range of Motion Tracking” graph useful or helpful to keep track of patient recovery? (4 - very helpful)	0	0	1	1	3	4	1
Is the “Pain Levels Trends” graph useful or helpful to keep track of patient recovery? (4 - very helpful)	0	0	0	3	2	3	1
Is the “Compliance Score” useful or helpful to keep track of patient recovery? (4 - very useful)	0	0	0	0	5	4	0
Is the printout summary of patient progress useful or helpful by giving a concise and comprehensive view of the patient’s progress? (4 - very helpful)	0	0	0	0	2	4	0
						**Yes**	**No**
Would you consider using the *interACTION* device to manage rehabilitation again in the future?						6	0

**Table 7:** Patient interviews (n = 7).

	Patient #s
**Affirmations**	
Easy to use	3,6
Feedback and/or media useful	1,3,4,7
Hastened rehab	2,5
Helpful when working	1,3,7
Gave motivation/accountability	1,2,4,5,6,7
Other affirmation	1,2,3,5,6
**Criticisms**	
Calibration/counting errors	3,4,5,6,7
Crashes or system failure	1,3,5,7
Demotivating when not working	5
Difficult to put on	2
Fit poorly	5,7
Learning curve	3
Limited feedback	1,7
Difficult to set up	6,7
Other criticism	3,5,6
**Suggestions**	
Allow provider to change order of exercises	5
Make setup faster (procedural)	3,7
Improve the harness/straps (fit, stability, or comfort)	1,5,7
Improve the user interface	1,3,5
Create better instructions	1,2
Include more ranges of motion	7
Include more exercises	4
Make exercises progressive for therapy	4
Other suggestions	7

**Table 8:** Provider interviews (n = 9).

	Provider #s
**Affirmations**	
Integrates well into clinic	1,8,9
Easy to modify exercises	10,11
Feedback is good for the patient	4,9,10
Provides motivation/accountability	4,5,7,8,10
Improves patient outcomes	1,10
Portal is easy to use	1,8
System is self-contained	10
Reports and tracking are useful	5,6,7,9,10
Exercises are useful	8,10
Other affirmation	1,6,10
**Criticisms**	
Calibration/count errors	1,5,6,8,9,10
Difficult to change order of exercises	8
Not enough exercises included	6,7
Portal layout is confusing	10
Patient difficulty and frustration	4,5,6,8,10
Setup (initial/clinical) is difficult	4,5,10
Other criticism	6,10,11
**Suggestions**	
Simplify the system for patient	7,10
Give audio/voice feedback to patient	1,6
Count “partial” exercises	6
Increase accuracy	5,10
Include more exercises	10
Sense more ranges of motion	6,10
Improve harness, make sleeve	5
Create tutorials	10
